# Mobile Robots for In-Process Monitoring of Aircraft Systems Assemblies

**DOI:** 10.3390/s22093362

**Published:** 2022-04-27

**Authors:** Marc Auledas-Noguera, Amer Liaqat, Ashutosh Tiwari

**Affiliations:** 1 Department of Automatic Control and Systems Engineering, University of Sheffield, Sheffield S1 3JD, UK; a.tiwari@sheffield.ac.uk; 2 Assembly Innovation & Development, Airbus, Broughton CH4 0DR, UK; amer.liaqat@airbus.com

**Keywords:** automatic inspection, aerospace assembly, mobile robotics, depth camera

## Abstract

Currently, systems installed on large-scale aerospace structures are manually equipped by trained operators. To improve current methods, an automated system that ensures quality control and process adherence could be used. This work presents a mobile robot capable of autonomously inspecting aircraft systems and providing feedback to workers. The mobile robot can follow operators and localise the position of the inspection using a thermal camera and 2D lidars. While moving, a depth camera collects 3D data about the system being installed. The in-process monitoring algorithm uses this information to check if the system has been correctly installed. Finally, based on these measurements, indications are shown on a screen to provide feedback to the workers. The performance of this solution has been validated in a laboratory environment, replicating a trailing edge equipping task. During testing, the tracking and localisation systems have proven to be reliable. The in-process monitoring system was also found to provide accurate feedback to the operators. Overall, the results show that the solution is promising for industrial applications.

## 1. Introduction

Currently, the installation of systems on large aerostructures is manually fulfilled by skilled operators. The dexterity required for the assemblies, and the constrained access to certain areas, has limited the use of robotics. Nonetheless, there is significant interest in introducing new technologies onto the shop floor to improve the productivity, traceability, and quality control of these installations, as explained by Bogue in [1].

Aircraft systems are usually installed using fixings, such as clamps or ties, that mostly rely on the operator’s skill for compliance. This could lead to some misalignments that may need rework in later stations, causing delays on the assembly line. Additionally, if a part is incorrectly placed and requires adjustment it can cause other correctly placed parts to also be removed and reinstalled again because system installations are often sequential tasks.

**Figure 1:**
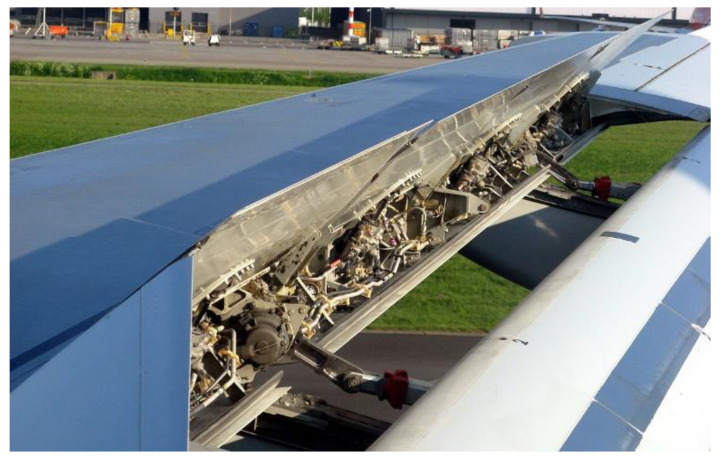
Detail of the numerous systems installed on the trailing edge of an A320 wing. Photograph by Annom, distributed under a public domain licence [3].

The trailing edge of an aircraft’s wing is an area of special interest because of the numerous systems concentrated there, as seen in Figure 1. For this reason, it has been used as a case study in this article. Many control surfaces are attached onto the rear spar, requiring an extensive set of hydraulic pipes, as described by Moir and Seabridge in [2]. Additionally, numerous cable bundles from electric systems on the wings are also routed through the trailing edge of the aircraft. These pipes and cables can extend through several metres on the rear spar of the aircraft. Many of these elements also need to be manually inspected after being installed.

For all these reasons, an automatic inspection system that could provide instant feedback to the workers about the state of the assembled systems would be highly beneficial. It could be used to ask operators to rework any misaligned parts before these proceed to posterior stations, avoiding production delays down the line.

The article is organised as follows. After the introduction, the related work is reviewed in Section 2. Then, a summarised overview of the proposed solution is provided in Section 3. The detailed explanation of the system is presented in Section 4. Section 5 describes the experimentation setup and analyses the results obtained using the proposed methods. Finally, Section 6 concludes the article and gives recommendations for future work.

## 2. Related Work

Several research articles have studied the use of sensing devices to monitor the progress of assemblies and provide feedback to the operators. As reviewed by Menolotto et al. in [4], motion-capture technology has been used to improve quality control or assembly processes for different types of industries. These systems usually monitor the state of the worker and the workpiece and then provide some guidance to the operator, as analysed by Tiwari et al. in [5].

Depth cameras, also known as RGB-D sensors, have been employed in many different applications to capture data about the worker and the components. For example, Prabhu et al. [6] used depth cameras to supervise operators during composite layup tasks. Chen et al. [7] utilised an RGB-D sensor and deep learning algorithms to monitor the manufacture of a small gear reducer. Costa et al. [8] deployed a depth camera to monitor the packaging of highly customizable gift boxes. Tarallo et al. [9] used a depth camera to monitor the assembly of small parts onto a thermoplastic component for a commercial vehicle. Faccio et al. [10] developed a system based on an RGB-D sensor to support operators assembling water pumps. Oyekan et al. [11] employed a depth camera to monitor packaging and electronics assembly operations. In the aerospace sector, depth cameras have also been employed for inspection before assembly operations. For example, Maiolino et al. [12] utilised an RGB-D sensor to inspect the correct placement of a bracket on a trailing edge rib before sealing it with an industrial robot. Maiolino et al. [13] would later expand on this application using the depth camera to inspect and estimate the pose of the components to modify the program of the industrial robot that applies sealant to the parts.

The previously presented studies showed great promise monitoring manual assemblies but focused on small-sized products and applications. However, these approaches could not be directly applied to monitor the installation of systems in a trailing edge because of the length of the wing and the limited field of view and range of the depth cameras. For this application, multiple sensors or a mobile system would be required.

The use of multiple sensors has been tested in tasks when larger objects need to be monitored. For example, Macknojia et al. [14] developed a calibration methodology for multiple RGB-D sensors and used it to scan a car with five Kinect cameras. Prabhu et al. [15] deployed two depth cameras to track and automate the installation of a wheel on an automobile using an industrial robot. This work was later continued by Prabhu et al. in [16]. The results shown were promising, with a system capable of tracking the wheels over the entire workstation length of 2.5 m with a small error, below 4 mm.

To monitor the systems installed on the trailing edge, numerous depth cameras could be used. However, many RGB-D sensors would be required to achieve accurate results and avoid occlusions from the structural parts of the wing. Additionally, these would be located far away from the assembly to not obstruct the workers. Overall, the installation of a multiple sensor rig in an aircraft assembly line would be complex.

Because of this, a mobile system would be preferable for the trailing edge of an aircraft. An autonomous mobile robot could follow the operator, keeping the inspection sensor at an optimum distance while avoiding occlusions from structural parts of the wing. Also, the system could carry the necessary parts or tools for the installation, allowing the operator to focus only on the assembly. Finally, another advantage is that trailing edge systems are installed in different stations and a single mobile system could be easily deployed at different stages of the assembly.

In recent years, there has been a growing interest in mobile robots for inspection of large-scale structures, including aircraft, as reviewed by Almadhoun et al. in [17]. An early example of this was the Air-Cobot, a project that started in 2013 and was aimed at automating preflight inspections at airports [18,19]. The system was composed of a mobile robot and, over the years, different sensors were tested for the inspection of the aeroplanes, including cameras, as presented by Leiva et al. in [18], and 3D scanners, as described by Bauda et al. [20] and Jovančević et al. [21]. A method to avoid obstacles during inspection was also developed by Leca et al. [22].

More specifically, mobile robots have also been applied in the aircraft manufacturing industry. Zhou et al. [23] used a mobile manipulator to apply sealant on large-scale components and then inspect these parts with a laser triangulation sensor as end-effector. Abdallah et al. [24] developed an inspection system that used 2D images, captured by a mobile manipulator or handheld tablet, to check the parts installed on an aeroplane engine. This work would be later expanded by Abdallah et al. [25] using a 3D scanner to inspect cables installed on aircraft engines.

These mobile systems can inspect large-scale aircraft components; however, they are focused on autonomous quality control of the parts, with little human involvement. Ideally, for system installations, feedback would be provided during the manual assembly process. This would allow trained operators to rework any misaligned parts before they proceed to later stations.

Inspired by this previous work, this paper presents the design and implementation of a mobile robot for the in-process monitoring of aircraft systems assembly. The aim of the proposed solution is to combine the previously presented online process-monitoring methods with a mobile robot, capable of moving along large-scale aerospace structures and being used in multiple assembly stations. The main novelty of the proposed system lies in its autonomous capabilities, which allow the mobile robot to follow the operator while installing the parts to provide feedback in real time.

## 3. System Overview

**Figure 2:**
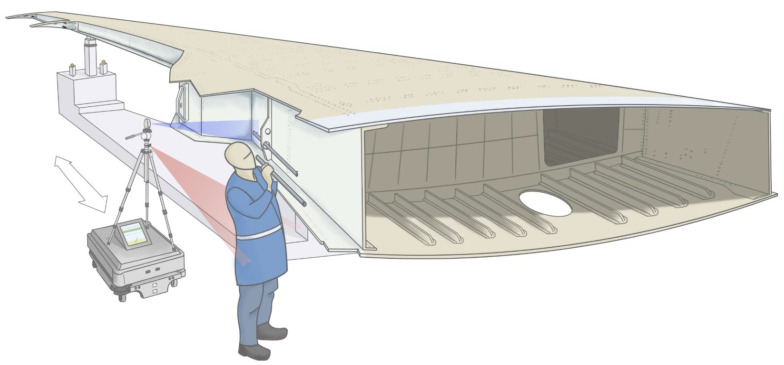
An illustration of the mobile robot tracking the operator and providing feedback about the systems installed on the trailing edge of the aircraft. Aircraft wings are equipped at several stations and are moved from one to another on wing trolleys. A mobile robot could be used to inspect wings at several stations, requiring no infrastructure modifications.

The system is built on top of an MiR200 mobile robot, as shown in Figure 2. Overall, the system is controlled with a laptop running Ubuntu 18.04 and ROS Melodic Morenia.

A tripod is placed on top of the platform to carry the sensors. An Intel RealSense L515 lidar camera or a D435 depth camera have been tested for the inspection of the assembly. These sensors have been placed on the tripod and used to inspect the height and depth of the systems. Based on the measurements obtained by the sensors, the system installation is classified as correctly assembled or not.

A Lepton 3.5 microbolometer thermal camera is used to track the operator. This sensor is placed on a 3D-printed mount on the tripod. The algorithm is designed to follow human body temperature, moving the mobile robot forward or backward using proportional control. A bang-bang controller is used to keep the orientation of the mobile platform constant, moving in parallel to the trailing edge. The MiR200 localisation system is used to know the position that is being monitored in length.

The screen of the laptop is used to provide online feedback during the assembly process. Height and depth data are compared to a model to provide guidance to the operator.

Once the operation has been completed, the MiR200 navigation system can be used to send the robot to another station or to the starting position again to repeat the task later.

A video demonstration of the system is available at the Supplementary Materials (Video S1) https://youtu.be/aqVmEy09ctk (accessed on 13 February 2022).

## 4. Methods

This section provides further information on the methods of the proposed solution and has been divided into three parts. First, the elements used to follow and localise the operator are presented. The second part describes the inspection sensors and algorithms. Finally, the proposed feedback mechanism is also discussed.

### 4.1. Operator Tracking and Localisation

The first part of the proposed solution are the sensors and algorithms to track and localise the operator.

#### 4.1.1. Worker Tracking

**Figure 3:**
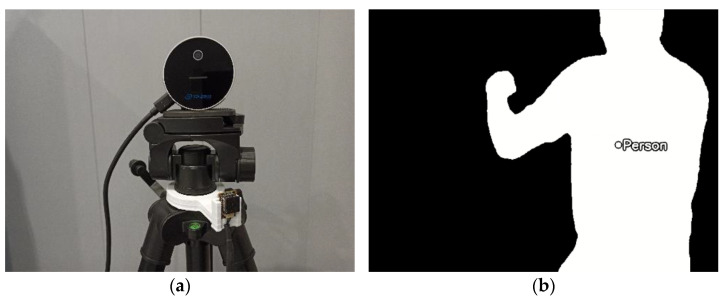
(**a**) Detail of the sensor arrangement. An Intel RealSense depth camera is located on top of the tripod and used for the inspection. Below, on a white 3D-printed support, the Lepton 3.5 provides thermal data to follow the operator; (**b**) binarized temperature map provided by the thermal camera, used to follow the operator installing the systems on the trailing edge of the aircraft.

A Lepton 3.5 is used to follow the operator; this sensor is an uncooled and radiometric-capable microbolometer, shown in Figure 3a. It can sense longwave infrared radiation, from 8 µm to 14 µm wavelengths, effectively capturing true temperature values. The output of this sensor is a 160 × 120 pixel temperature map, with a 57° horizontal field of view, 71° diagonal field of view, and a framerate of 9 fps [26].

The main reason to select a thermal camera for the tracking was the restriction on collecting a dataset that showed workers with regular cameras because of privacy concerns. In addition, because of the proximity to the operator, using pretrained models was difficult. Thus, a low-cost thermal camera was seen as a good option that would not require a dataset to follow the worker.

To track the operator, the algorithm uses the radiometric data. First, the temperature array is filtered with an upper and lower heat threshold, segmenting the operator from the background. The output is a binarized image, with the worker highlighted in white while the background is black. Then, the centre of gravity of the white region is calculated, as depicted in Figure 3b.

Depending on the position of the centre of gravity, the mobile robot is commanded to advance or move back, following the operator along the trailing edge of the aircraft. If the worker is too close to the thermal camera the algorithm will automatically stop the robot. Additionally, the laser safety sensors of the MiR200 are always active, preventing any collisions. Proportional control is employed to track the position of the person, with a maximum velocity of 0.3 m/s. The maximum speed and the safety systems have been implemented to comply with ISO 3691-4:2020 [27]. In addition, when moving at low speeds the system should be more stable, decreasing the tilting of the tripod and improving the measurements from the depth camera. Finally, to ensure that the sensor is perpendicular to the system being installed, a bang-bang controller has been implemented to move the robot in parallel to the trailing edge.

The control commands for the MiR200 are sent using the mir_driver developed by researchers from the German Research Center for Artificial Intelligence [28].

#### 4.1.2. Robot Localisation

Localising the mobile robot is important to correlate the monitoring data to a specific section of the wing. The combination of the inspection and localisation measurements could be stored and used for traceability, allowing a more detailed processing of the data coming from the sensors.

**Figure 4:**
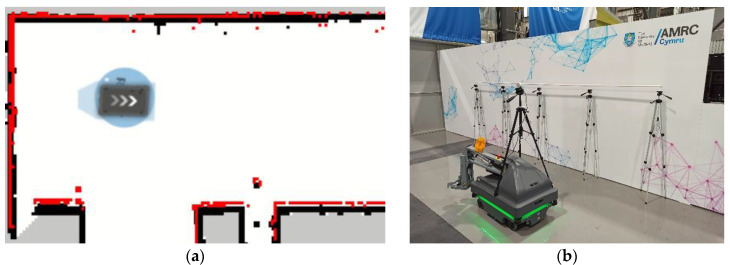
(**a**) Map of the operation area. Black pixels are mapped obstacles, red pixels are current sensor readings, and the starting position for the experiments is depicted as a blue circle, with the robot localised on top of it. (**b**) Image of the operation area represented on the map.

To know the robot’s pose on the trailing edge of the aircraft, the localisation system of the MiR200 was used. Firstly, a map of the area of operations was generated using the simultaneous localisation and mapping system of the MiR200, as seen in Figure 4. Then, during operations, the mobile robot localises itself with an adaptive Monte Carlo localisation algorithm utilising Sick S300 laser scanners, wheel encoders, and an IMU. The output of this system is the pose of the robot.

### 4.2. Monitoring Sensors and Algorithm

The second element of the system consists of the sensors and the algorithm used to estimate if the system has been correctly installed. Two depth cameras have been tested and could be used for the inspection of the assembly: an Intel RealSense D435 depth camera and an Intel RealSense L515 lidar camera.

The D435 depth camera consists of an RGB camera, an infrared pattern projector, and two infrared cameras. To obtain depth estimates, the D435 uses an approach known as active stereoscopy. The infrared projector generates a pattern of identifiable features that the two infrared cameras use to triangulate the distance. The outputs of this sensor are high-resolution RGB images and depth maps with a resolution of 1280 × 720 pixels, with a 87° × 58° field of view and a framerate of up to 90 fps [29].

The L515 lidar camera, on the other hand, consists of an RGB camera, an IMU, and an infrared laser emitter and receiver coupled with a MEMS mirror [30]. To estimate the depth of the scene, this sensor uses a light detection and ranging system coupled with a MEMS mirror to scan the scene. The output of this sensor are high-resolution RGB images and depth images with a resolution of 1024 × 768 pixels, a 70° × 55° field of view, and a framerate of 30 fps [31].

Even though each depth camera has a different operating principle, both sensors have similar depth resolutions and fields of view. The frame rate is lower for the L515 lidar camera; however, the mobile robot will be moving at a slow pace and this should not be an issue.

These depth cameras have been placed on top of the tripod on the mobile robot. Using the tripod cranks, the height has been adjusted to bring the sensors close to the same height as the systems being installed. The reason to place the depth cameras in this position is to improve the accuracy of the system, reducing the negative impacts of any optical aberrations on the lenses of the sensors.

Then, the inspection algorithm analyses each image provided by these RGB-D sensors. The depth maps generated are defined as two-dimensional functions *f*(*x*,*y*), where *f* is the depth in mm, while *x* and *y* are spatial coordinates. These images contain *M* rows and *N* columns, equivalent to the resolution of the camera. For every image received, the following algorithm is executed:

(1) Create a one-dimensional array: $depths=\left[f\left(\frac{M}{2},\;\frac{N}{2}-p\right),\;\cdots ,f\left(\frac{M}{2},\;\frac{N}{2}+p\right)\right]$, where 2*p* is the number of pixels inspected.
(2) Find the height of the installed system, *i*, in pixels. To do so, calculate the median index of all pixels with depth values within a threshold of the expected system depth. Then, convert the height of the system *i* to mm using a calibrated conversion value.
(3) Find the depth of the installed system, *r*, in mm. To do so, determine the approximation r≈z2−z1, where $z2=f\left(\frac{M}{2},\;i-25\right)$ and $z1=f\left(\frac{M}{2},\;i\right)$.

**Figure 5:**
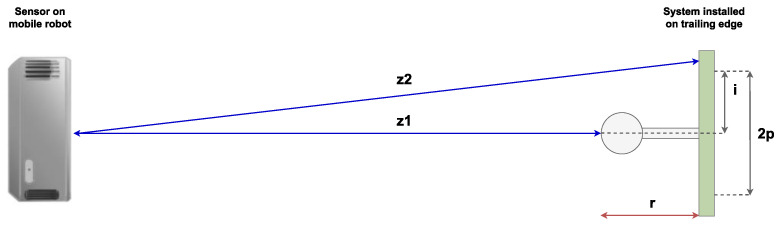
Side view of the system and the main variables used in the inspection algorithm. Image not to scale.

The main variables used in the inspection algorithm are shown in Figure 5 below.

### 4.3. Feedback Mechanism

Finally, the last element of the system provides online feedback to the operator. Fletcher et al. [32] developed a detailed analysis in 2020 about the requirements automated systems should fulfil to ensure worker satisfaction. In particular, for communication and interaction mechanisms, the survey found that the best way to provide feedback was using visual means. Auditory and visual cues were also found to be desirable, even though they were not preferred over only visual ones.

**Figure 6:**
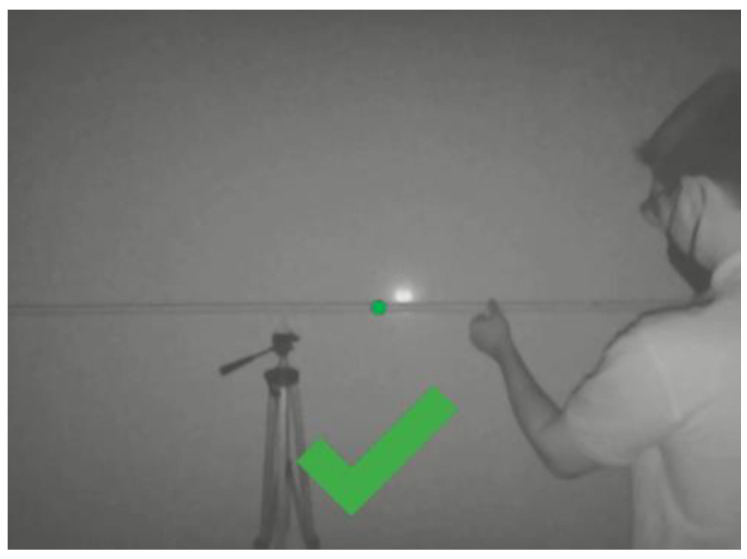
Example of the feedback provided by the system to the worker during operations.

Based on the results of this study, it was decided to implement a visual feedback mechanism. In this case, the depth map is displayed and an indication of the expected position of the system is shown on the vertical axis. Then, overlayed on this image, a tick or arrows are used to provide guidance to the operator. These visual cues indicate if the system is properly installed, or it needs to be moved vertically or in depth. Indications are based on the monitoring measurements and a set of conditional statements. An example of the feedback provided to the operator is shown in Figure 6 below.

This part of the system could be redesigned to better suit the needs of the operators on the assembly line. For example, auditory cues could be easily added if workers prefer not to look at the screen when performing the assembly. Similarly to the visual cues, a different sound could be used for every type of misalignment.

## 5. Results

The experimentation and results have been divided into three sections. Every part evaluates the performance of the previously presented methods in a laboratory environment. The first test analyses the reliability of the tracking system and the accuracy of the robot’s localisation. The second section examines the operating range of the two depth cameras and quantifies the accuracy of the real-time inspection on the move. Finally, the last part evaluates if the feedback provided to the operators is reliable. All datasets used and referenced in this section are publicly available at https://github.com/Auledas/monitoring_aircraft_systems_data (accessed on 13 February 2022).

### 5.1. Operator Tracking and Localisation Results

The aim of the first experiment is to determine the capacity of the system to localise itself while tracking an operator.

Reliable tracking is important to ensure that the robot is providing quick feedback to the worker. Accurate localisation of the robot is also required to correlate the inspection data, provided by the depth cameras, to a specific section of the trailing edge.

To test this, a trailing edge installation task was simulated in a laboratory environment. During assembly, operators progressively advance from one end of the trailing edge to the other. They stop at regular intervals to tighten the fasteners of the cables or hydraulic tubes.

**Figure 7:**
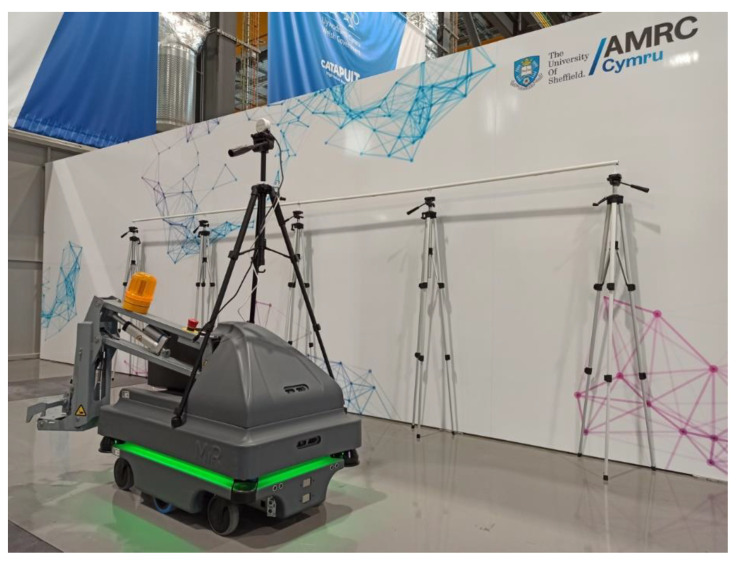
Experimental setup recreating an installation on a trailing edge.

To replicate this task, the experiment presents a 4 m long installation, shown in Figure 7. In this scenario, the person starts at one end of the installation and advances 25 cm in parallel to it. Then, to simulate the fastening, the person stops at that spot for 15 s. This is repeated through the entire system length, following the same movement pattern. In total, the experimental task takes 4 min to complete. Finally, the robot is autonomously sent to the starting pose to prepare for the next experiment.

**Figure 8:**
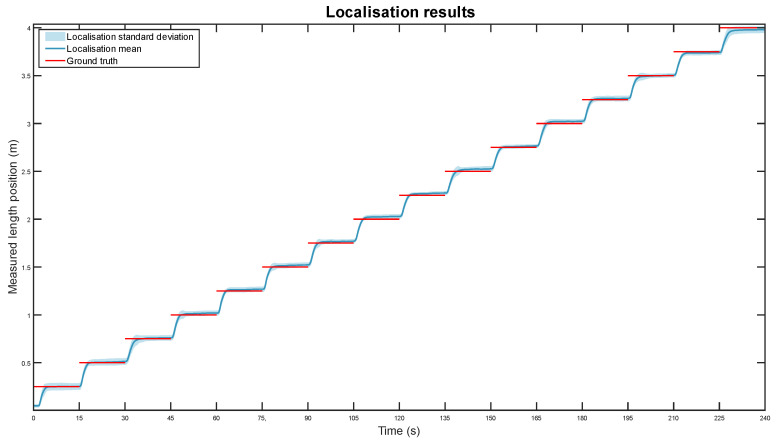
Measured location, in blue, compared to the actual robot position, in red, during the operator-tracking experiments.

A total of 30 experiments were conducted following the procedure described above. In total, the proposed system tracked a person for 2 h (120 m). The measurements from these experiments are grouped on dataset 1. The obtained graph is shown below, in Figure 8.

The line graph shows the robot’s average position in length over the duration of the task for the 30 experiments. The mean and standard deviation of the position measurements are displayed in dark and light blue, respectively. The ground truth, the expected position of the system if the robot correctly follows the operator, is also shown as red lines.

Overall, the mobile robot reliably tracked the operator throughout the entire task in all experiments. On average, it took between one to three seconds for the robot to follow the operator from one position to the next one, separated by 25 cm. The positioning error mean was quantified at 7.5 mm with a standard deviation of 18.6 mm, calculating the difference between the measurements and the ground truth after a settling time of 5 s for each position. Given the length of the component, this accuracy is considered acceptable.

The movement behaviour and the localisation accuracy in all displacements is very similar. The only one that has a different movement pattern is the first step, from second zero to fifteen. In this case, the robot started in a pose slightly ahead of the expected one, and it moved too slowly to go to its expected position. This issue, however, could be easily fixed by setting a more suitable starting pose behind the operator.

### 5.2. System Assembly Monitoring Results

To evaluate the performance of the online inspection system, three experiments were conducted.

The aim of the first experiment was to determine the most suitable sensor to inspect the systems installed on the trailing edge. The second test aimed at quantifying the accuracy of the depth measurements obtained by the inspection system. Finally, the last experiment checked the height accuracy of the monitoring sensor and algorithm.

#### 5.2.1. Operating Range

The objective of the first test was to find the sensor with the widest operating range to inspect common materials used on the systems installed in commercial aircraft.

This experiment compared two sensors: an Intel RealSense L515 lidar camera and an Intel RealSense D435 active stereoscopic camera. These depth cameras were used to detect four different cable channels and pipes. These parts, similar to components used in aircraft system installations, were:

- A 25 × 5 mm white PVC tower cable channel;
- A 25 × 25 mm grey PVC square cable channel;
- A ⌀20 mm white PVC circular pipe;
- A ⌀16 mm aluminium circular pipe.

**Figure 9:**
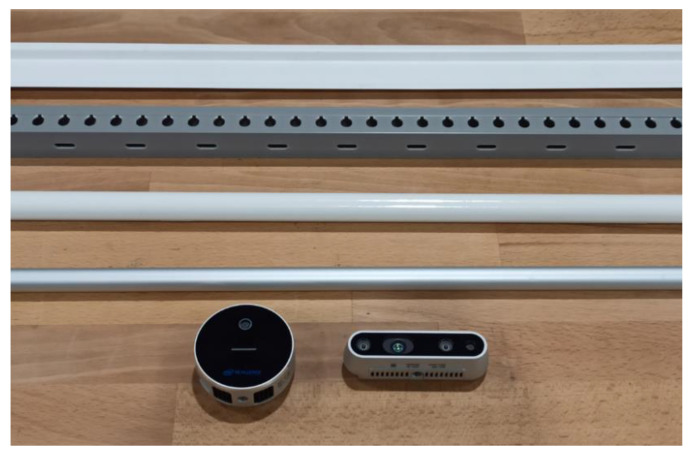
The inspected parts, above, and the sensors, below, used during experimentation.

The sensors and inspected parts can be seen below, in Figure 9.

A total of eight experiments were conducted. During the tests, one of the sensors was placed on top of a tripod. Then, a part was placed perpendicularly in front of it with a starting separation of 500 mm. Every 15 s the tripod was moved back 100 mm until reaching a distance of 2000 mm from the component. The data from these experiments can be found in dataset 2. The detection-range results are attached below in Table 1.

The table compares the capabilities of the L515 lidar camera and the D435 active stereo camera to detect the materials shown in Figure 9.

Overall, the L515 lidar camera offered a much wider operating range in all cases. Cable channels were detected by both the L515 and the D435. The lidar camera offered a wide operating range, reaching up to 2 m. By contrast, the D435 could not detect the cable channels at distances greater than 700 mm.

The pipes were more difficult to inspect for the two sensors. The L515 could reliably detect both tubes, even though the range was not as wide as with the cable channels. On the other hand, the D435 was not able to detect the aluminium pipe and could only detect the PVC pipe up to a separation of approximately 600 mm.

As a result, it was decided to proceed with the experiments using the L515 sensor.

#### 5.2.2. Depth Inspection

The second monitoring experiment focused on quantifying the depth accuracy of the system on the move.

The depth of the systems being installed on an aircraft needs to be within a certain tolerance. These limits ensure that the components are not loosely fastened and that they will not obstruct parts installed later during the process. For these reasons, it is crucial to evaluate the accuracy of the system. In this case, as a proof of concept, the tolerance limit was set at ±3 mm of the expected depth position.

The experiment was conducted in the environment shown in Figure 7 and with the same moving patterns as described in Section 5.1. In this case, the ⌀20 mm white PVC circular pipe was placed 200 mm away from the wall in front of the sensor. Due to inaccuracies during the placement, the parts could be within ±2 mm of the mark. The mobile robot moved in parallel to the wall, with a separation of approximately 1.2–1.3 m, within the operating range of the L515.

**Figure 10:**
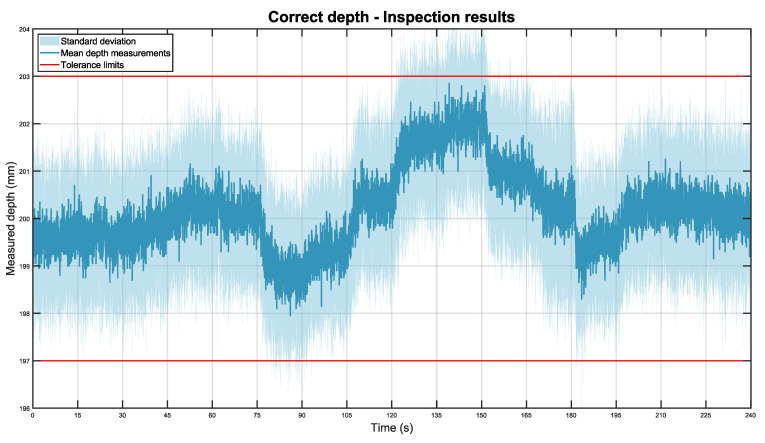
Mean depth position of the pipe in dark blue, with its standard deviation in light blue. The tolerance limits are shown in red.

Using this setup, 20 experiments were conducted. In total, the system monitored the installation for 1 h and 20 min (80 m). These measurements are included in dataset 3. The obtained results are attached in the graph below in Figure 10.

The graph illustrates the average depth position of the pipe while the mobile robot is moving through the station for the 20 experiments. The depth mean is displayed in dark blue while the standard deviation is shown as a light blue area. The tolerance limits, the depth distances that the pipe should not exceed, are displayed as two red lines.

In general, the system was able to detect the pipe throughout the station with high accuracy and repeatability. The total depth mean for all experiments was 200.18 mm with a standard deviation of ±0.84 mm. Nonetheless, as it can be seen in the graph, the mean is not constant for all positions of the robot. These variations are caused by operator inaccuracies during the placement of the part. However, overall, the system was capable of correctly detecting that the part was within the tolerance limits in all positions.

A remarkable feature of the system is the inspection repeatability. Given that the inspection sensors are placed on a mobile robot, the obtained standard deviation values can be considered excellent.

**Figure 11:**
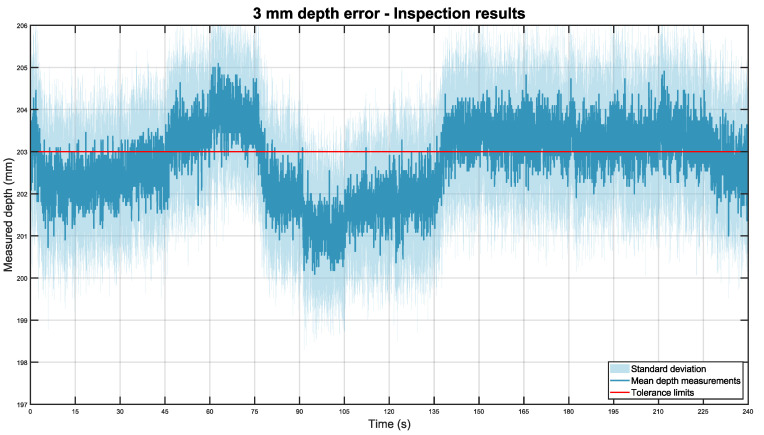
Mean depth position of the pipe placed 3 mm farther than expected in dark blue, with its standard deviation in light blue. The tolerance limits are shown in red.

This experiment was replicated with the pipe placed 3 mm and 5 mm farther than expected. In each case, the experiment was repeated 11 times for a total of 44 min of inspections (44 m). The data from these experiments are included in datasets 4 and 5. For the system installed at a distance of 203 mm, Figure 11 was obtained.

Overall, the obtained results are similar to those of the correct placement inspection, with high accuracy and repeatability. In this case, the total depth mean was 202.77 mm with a standard deviation of ±0.88 mm.

**Figure 12:**
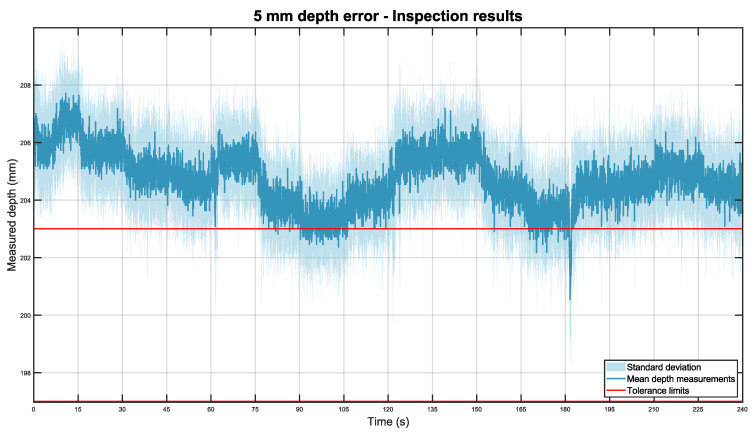
Mean depth position of the pipe placed 5 mm farther than expected in dark blue, with its standard deviation in light blue. The tolerance limits are shown in red.

For the pipe installed 5 mm farther than expected, Figure 12, below, was obtained.

Again, the results were consistent with previous experiments. In this case, the total depth mean was 204.81 mm with a standard deviation of ±0.92 mm.

#### 5.2.3. Height Inspection

Finally, the third inspection experiment focused on analysing the height accuracy of the system while moving. The height position was evaluated because it is also a crucial factor for the correct installation of aircraft systems. Again, as a proof of concept, the tolerance limits were set at ±3 mm of the expected position.

The tripod with the sensor and the inspected pipe were both placed at a height of 1.4 m during the tests. As explained in Section 4.2, the height of the system is provided in pixels and needs to be calibrated to obtain the value in millimetres. In this case, the system was calibrated at a distance of 1.25 m. At that range, every pixel in the vertical axis was equivalent to 1.8 mm. During inspection, a range of 20 pixels around the expected position of the system was analysed, a total of 36 mm.

The dataset used in this analysis was collected at the same time as the correct depth inspections, composed of 20 experiments, in dataset 3. Similarly to the depth inspections, the monitored parts could be within ±2 mm of the expected position.

**Figure 13:**
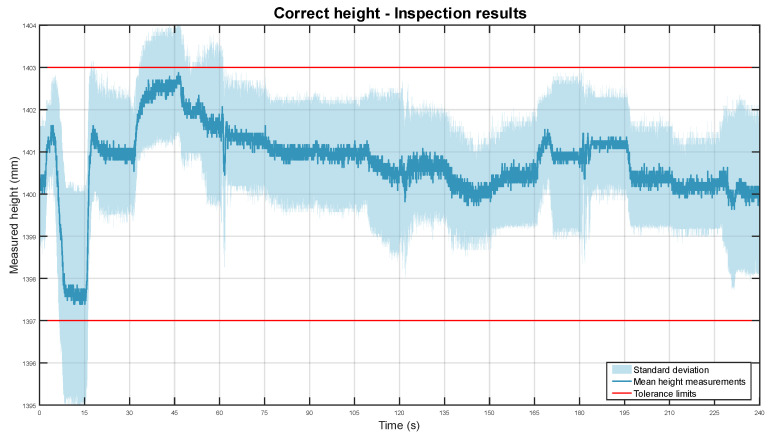
Mean height position of the pipe in dark blue, with its standard deviation in light blue. The tolerance limits are shown in red.

The graph obtained from the data is attached below in Figure 13.

The line graph shows the average height position of the system for all 20 experiments. The height mean is shown in dark blue and the standard deviation is represented as a light blue area. The ±3 mm height tolerance limits are presented as red lines.

Overall, the algorithm correctly monitored the height of the installed system. The total height mean was 1400.75 mm with a standard deviation of ±0.87 mm. In contrast with the depth inspections, the mean is less dispersed and it has a more significant offset at 0.75 mm. This is caused by the lower resolution on the vertical axis, which reduces measurement granularity and magnifies inaccuracies of the system placement. Nonetheless, given the tolerances, it can still effectively monitor the vertical alignment of the component.

It is important to highlight an outlier section just at the start of the inspection, approximately from second 5 to 15. The height values there are well below those around them. This was caused by an irregularity on the floor where the mobile robot was moving. In that zone, a panel is ~4 mm above the rest of the flooring. This can be seen in Figure 4b and Figure 7 as a darker area on the floor behind the robot. In consequence, the pipe was detected lower than in other parts of the setup. To solve this problem, the localisation of the robot could be used to adapt the expected height position of the system.

**Figure 14:**
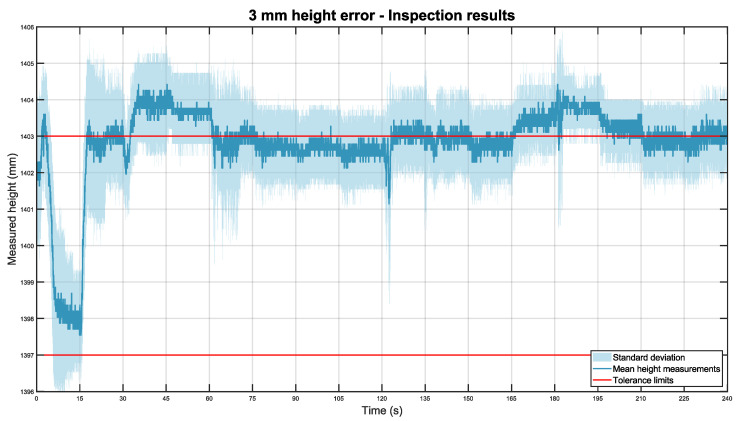
Mean height of the pipe placed 3 mm higher than expected in dark blue, with its standard deviation in light blue. The tolerance limits are shown in red.

This experiment was reiterated with the pipe placed 3 mm and 5 mm higher than standard. These experiments were repeated 11 times in every scenario, resulting in 44 min of inspections (44 m). The measurements are included in datasets 6 and 7. Below, in Figure 14, the graph when the pipe is 3 mm higher is included.

In general, the line graph obtained closely resembles the previous figure, with similar accuracy and repeatability. In this case, the total height mean was 1402.83 mm with a standard deviation of ±1.11 mm.

**Figure 15:**
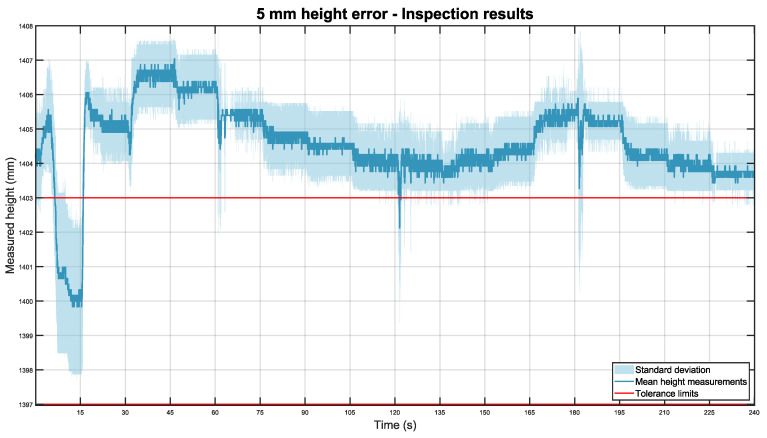
Mean height of the pipe placed 5 mm higher than expected in dark blue, with its standard deviation in light blue. The tolerance limits are shown in red.

When the pipe was 5 mm higher, Figure 15 was obtained:

The last inspection experiment was also consistent, with a total height mean of 1404.58 mm and a standard deviation of ±1.16 mm.

### 5.3. Feedback Evaluation

Finally, the last section of the results analyses the feedback provided to the operator during assembly.

Consistently accurate feedback is crucial for the acceptance of this technology on the shop floor. Unreliable indications would discourage workers from using the system.

To evaluate the feedback cues, each indication provided during assembly has been recorded and compared to the actual position of the system. Datasets 3, 4, 5, 6, and 7 have been used for this analysis. These results have been divided into two tables, one for depth and the other one for height indications.

#### 5.3.1. Depth Feedback Evaluation

The first analysis focuses on the depth feedback displayed to the operator. The feedback is provided using conditional statements based on the measurements. If the value is between 197 and 203 mm, both inclusive, the cue shows a tick, indicating the system to be within tolerance. If the value is higher than 203 mm, the feedback suggests moving the system closer to the trailing edge. Finally, measurements lower than 197 mm prompt the operator to move the pipe farther away from the trailing edge.

Datasets 3, 4, and 5 are used in this study. From each dataset, 11 experiments are analysed. Each test is composed of 4 min of depth measurements. Each time a depth value is received by the system, the indication on the screen is updated. Since the L515 lidar camera has a framerate of 30 fps, every second 30 cues were shown on the screen. Thus, for each dataset a total of 79,200 feedback indications were displayed.

Below, in Table 2, the real depth position is contrasted with the feedback provided to the operator.

Overall, the feedback displayed to the operator was mostly correct for all three datasets.

When the pipe was centred, in dataset 3, the feedback provided was that the system was within tolerance 96.8% of the times. This is an excellent result that correctly confirms that the system is properly placed.

In dataset 4, the pipe is moved 3 mm away from the trailing edge. In this case, due to positioning inaccuracies, some parts of the pipe may be within tolerance while some other sections may be out of specifications. As expected, in some sections the feedback provided was that the system was within tolerance (66.7%), while in other parts the indications were that the pipe was installed too far away (33.3%).

Finally, when the pipe is 5 mm away from its expected position, the feedback provided shows 79.5% of the time that the system is too far from the trailing edge. This is a good result, warning most of the time that the pipe has been incorrectly installed.

#### 5.3.2. Height Feedback Evaluation

The second feedback study evaluates the height indications provided to the operator. Again, a conditional statement model is used to provide feedback to the worker. If the system is estimated to be a height from 1397 mm to 1403 mm, both inclusive, the feedback shows the system is within tolerance. If the measurement is above 1403 mm or below 1397 mm, then the feedback indicates that the pipe needs to be repositioned.

Datasets 3, 6, and 7 are used in this evaluation. Similarly to the previous section, for each dataset a total of 79,200 feedback indications were provided to the worker.

Table 3 below contrasts the actual height position of the system with the feedback displayed to the operator.

In dataset 3, the system is properly centred in height and the feedback provided was correct 96.7% of the times. This is a robust result, demonstrating that the monitoring system is reliable.

Then, the pipe was placed 3 mm above its expected location in dataset 6. At the tolerance limit, the expected result was to have the feedback divided into two categories. Indeed, the analysis shows that there were indications that the system was too high 63.8% of the times and within tolerance 34.9% of all instances.

Finally, when the system is installed 5 mm higher than its standard position, the feedback clearly highlights a problem. In this case, 96.3% of the times the indications show that the pipe is placed above tolerance.

Overall, as in the feedback provided for the depth measurements, the results were excellent. Thus, the indications given to the worker are reliable and should be useful preventing errors during assembly.

## 6. Conclusions

This article presents an in-process monitoring based on a mobile robot to ensure quality control during aerospace equipping processes. The proposed solution has several characteristics that have been tested in a laboratory environment.

First, a thermal camera is used to track the operator. During testing this approach has been robust and the mobile robot correctly followed the operator in all cases. To localise the inspection position, 2D lidars are used. The localisation results were accurate and provided enough detail to correlate inspection data with a certain position. However, the tracking and localisation system has not been tested in a factory environment. Because of this, the tracking system should be further tested to ensure it can correctly track the operator in a station with multiple workers. Similarly, the localisation capabilities should be further tested in a dynamic environment, where obstacles could reduce its accuracy.

Second, an inspection system using a depth camera is used to perform in-process monitoring. The results showed that the proposed monitoring approach would work with most cable channels and hydraulic pipes in a wide distance range. During moving inspection tests, at a distance of approximately 1.2 m, the height and depth measurements obtained were consistently very accurate, with little deviation.

Finally, online feedback is provided to the operators to ensure that the system is correctly installed. During experimentation, the indications were found to be predominantly correct and reliable.

Overall, the proposed solution is promising and could help improve current aerospace manufacturing. Future research directions could aim at inspecting difficult-to-reach areas, such as the interior of aircraft wings, using mobile manipulators. Another line of research could tackle more complex system geometries in tighter areas, correlating localisation data and wing structural features with the expected system pose.

## Figures and Tables

**Table 1:** Approximate detection range of the inspection system depending on the materials and sensors used. The materials are arranged in the same order as seen in Figure 9.

Materials	L515 Lidar Camera Detection Range (mm)	D435 Active Stereo Camera Detection Range (mm)
White PVC cable channel 25 × 5 mm	~500–2000	~500–700
Grey PVC cable channel 25 × 25 mm	~500–2000	~500–700
White PVC pipe ⌀20 mm	~500–1600	~500–600
Aluminium pipe ⌀16 mm	~500–1200	No detection

**Table 2:** Table evaluating the depth position feedback provided to the operator against the actual depth of the system. Correct feedback is highlighted in green. Incorrect feedback is highlighted in red.

	Feedback Provided		
Real Depth Position	Too Shallow	Within Tolerance	Too Deep
Centred	2.2%	96.8%	1%
3 mm depth error	33.3%	66.7%	0%
5 mm depth error	79.5%	20.5%	0%

**Table 3:** Table evaluating the height position feedback provided to the operator against the actual depth of the system. Correct feedback is highlighted in green. Incorrect feedback is highlighted in red.

	Feedback Provided		
Real Height Position	Too High	Within Tolerance	Too Low
Centred	0%	96.7%	3.3%
3 mm height error	63.8%	34.9%	1.3%
5 mm height error	96.3%	3.7%	0%

## Data Availability

The analysis scrips and data used in the results section can be downloaded at https://github.com/Auledas/monitoring_aircraft_systems_data (accessed on 13 February 2022).

## Supplementary Materials

The following supporting information can be downloaded at: https://youtu.be/aqVmEy09ctk, Video S1: Mobile Robots for In-Process Monitoring of Aircraft Systems Assemblies.
